# Guidelines for Best Practice in the Audiological Management of Adults Using Bimodal Hearing Configurations

**DOI:** 10.1097/ONO.0000000000000011

**Published:** 2022-06-24

**Authors:** Jourdan T. Holder, Meredith A. Holcomb, Hillary Snapp, Robert F. Labadie, Jantien Vroegop, Christine Rocca, Mohamed Salah Elgandy, Camille Dunn, René H. Gifford

**Affiliations:** 1 Vanderbilt University Medical Center, Nashville, TN;; 2 University of Miami, Miami, FL;; 3 Medical University of South Carolina, Charleston, SC;; 4 Erasmus Medical Center, Rotterdam, The Netherlands;; 5 Guy’s and St. Thomas’ Hearing Implant Centre, London, United Kingdom;; 6 University of Iowa, Iowa City, IA.

**Keywords:** Bimodal, Cochlear implant, Hearing aid

## Abstract

Clinics are treating a growing number of patients with greater amounts of residual hearing. These patients often benefit from a bimodal hearing configuration in which acoustic input from a hearing aid on 1 ear is combined with electrical stimulation from a cochlear implant on the other ear. The current guidelines aim to review the literature and provide best practice recommendations for the evaluation and treatment of individuals with bilateral sensorineural hearing loss who may benefit from bimodal hearing configurations. Specifically, the guidelines review: benefits of bimodal listening, preoperative and postoperative cochlear implant evaluation and programming, bimodal hearing aid fitting, contralateral routing of signal considerations, bimodal treatment for tinnitus, and aural rehabilitation recommendations.

## EXECUTIVE SUMMARY OF BIMODAL GUIDELINES

### Summary of Benefits of Bimodal Hearing

1. Although bimodal benefit is highly variable per individual listener (1–4), it offers significant benefits as compared to unilateral cochlear implant (CI) alone for speech recognition in quiet and noise for adults (1–3,5–8) and children (9–13).
2. Mean expected bimodal benefit is approximately 10- to 20-percentage points for speech recognition in quiet (1–3,5,6,14–16) and 10- to over 30-percentage points for both speech recognition in colocated noise (1–3,5,6,14,15,17) and in spatially separated noise (5,14).
3. Bimodal hearing offers sound quality benefits (more natural, full, pleasant) for speech and music (16,18–21) and less effortful listening compared to CI alone (16,22).
4. Most CI users with hearing thresholds below 90 dB HL will derive benefit from a hearing aid (HA) in the nonimplanted ear; however, if bimodal benefit is not demonstrated, a second CI should be considered (23).

### Summary of Preoperative CI Evaluation and Surgery

1. Providers should refer adults with hearing loss for a CI evaluation when they present with ≥ 60 dB HL 3-frequency pure tone average (PTA) and ≤ 60% unaided word recognition score in the better hearing ear (24).
2. CI candidacy evaluation should consist of standard audiometric testing, aided speech recognition testing using an appropriately fitted and verified HA, questionnaires, ear, nose, and throat (ENT) physician consult, radiologic imaging, and other referrals necessary for a specific patient (psychology, anesthesiology, speech-language pathology, etc.) (25).
3. CI surgery is typically completed in an outpatient setting with quick recovery time and minimal complications.

### Summary of Postoperative CI Fitting and Assessment

1. Realistic expectations for activation and postoperative improvement are important. Initial sound quality with the CI is variable but will typically improve over the first few months following initial activation with continued improvement in speech understanding over the first year of CI use (26).
2. Lower stimulation levels should be programmed according to manufacturer recommendations and verified using aided detection testing. Aided thresholds should be in the 20–30 dB HL range for 250 to 6000 Hz using frequency modulated (FM) warble tones to ensure appropriate access to speech sounds (27).
3. Upper stimulation levels should be optimized using electrically evoked stapedial reflex thresholds (eSRTs) to ensure that they are set appropriately (28–36) as behavioral measures such as loudness scaling are variable (37–40), and electrically evoked compound action potentials (eCAPs) are poor predictors of stimulation levels (29,41–48).
4. CI patients should return for follow-up to fine tune the CI programming and assess outcomes of the implanted device (23,25). Follow-up schedules vary but typically include 4–6 sessions in the first year of implantation (23,49).

### Summary of Bimodal HA Fitting

1. Real-ear verification of the aided response should be the standard of care when fitting the HA as adequate audibility is essential for HA benefit (50). No clear evidence was found on how certain choices in HA fitting formulas contribute to optimal bimodal performance. A standard fitting formula for severe hearing loss for which the target HA aided response is known, like National Acoustic Laboratories’ Nonlinear Fitting Procedure, Version 2 (NAL-NL 2) or Desired Sensation Level, Version 5 (DSL-5), is recommended (51–55).
2. Current evidence suggests that frequency lowering is not beneficial for bimodal CI users (51).
3. Synchronization of automatic gain control (AGC) between HA and CI is possibly beneficial (56–58); however, more research is needed to this topic. Currently, the matched-AGC approach is only clinically available with Advanced Bionics’ bimodal system. While CI clinicians can certainly alter the AGC in the HA software for other devices, there is no research to support this approach at present.
4. The additional value of interaural loudness balancing between HA and CI is not clear as it typically does not result in large deviations from the prescribed gain by the initial fitting formula (51–53,59–61).

### Summary of Evidence for Selecting a Contralateral Routing of Signal Device

1. Bilateral (62–67) or bimodal (68–70) stimulation should be prioritized unless otherwise contraindicated.
2. The greatest deficit for speech perception in noise in unilateral CI users is observed when the CI is masked by competing signals and the target is directed to the nonimplanted ear (62,64,71–73).
3. Contralateral routing of signal (CROS) is effective in improving the signal-to-noise ratio (SNR) at the deaf ear in unilateral CI users (62,64,71,72,74) and may improve hearing outcomes for targets in front of the unilateral CI listener (62,64,71,72,75).
4. Negative effects of CROS can be observed when competing signals (ie, noise) is transferred to the unilateral CI, although this is small in degree (64,71,72,75).
5. CI + CROS provides comparable benefit for lifting of head-shadow to bilateral CIs (64); however, localization is not improved by CI + CROS.
6. The most reliable method of validation CROS benefit is utilizing behavioral tests of head-shadow using measures relative to threshold to detect changes. Fixed speech-in-noise (SIN) measures of < +5 dB SNR may be too challenging for unilateral CI users and may underestimate CI + CROS benefit (64,72,75).

### Summary of CI + HA for Tinnitus Relief

1. Approximately 70%–80% of individuals suffering from tinnitus report improvement following CI (76–79); however, improvement cannot be predicted, so patients should be appropriately counseled regarding realistic expectations and supported with other appropriate therapies if necessary.
2. For some bimodal listeners, it is possible to integrate acoustic hearing with CI stimulation to further reduce troublesome tinnitus (14).

### Summary of Aural Rehabilitation

1. Not all adults require aural rehabilitation, but some have shown significant benefit from a structured aural rehabilitation approach (80). The current literature lacks a randomized clinical trial to unequivocally evaluate the effectiveness of aural rehabilitation.
2. There are many types of rehabilitation (clinician-led programs, self-guided at-home training, group, etc.) options for patients. Early evidence from HA users suggests that different types of therapy were equally effective (81).3. CI recipients likely require a personalized aural rehabilitation plan combining remote e-hearing health and in person opportunities to ensure that the therapy meets their goals and is sustainable for the treatment center and patient.

## INTRODUCTION

Cochlear Implants (CIs) are globally accepted as the standard of care intervention for adults with bilateral severe-to-profound sensorineural hearing loss (SNHL) (82). Supporting this stance is the fact that there are no published studies demonstrating a decrement in speech perception following CI for adults with bilateral severe-to-profound SNHL. Thus, in this population, “CIs unequivocally improve auditory and speech perceptual outcomes.”

It is estimated that in the United States alone, there are approximately 2.1 million adults aged 20+ years who have severe-to-profound SNHL (83). Most hearing loss can be at least partially remediated with HAs and hearing implants; however, it is estimated that only 14.2% of HA candidates over the age of 50 (84) utilize HAs and only 1%–7% of adult CI candidates pursue CI (85–87). At the time of preparation, there were no analogous data at a global level; however, the World Health Organization currently estimates there are over 432 million adults with disabling hearing loss, a figure expected to nearly double in just 30 years (88). Thus, as a field, we have much work to do to ensure that this growing population receives appropriate hearing healthcare and intervention and to broaden the application of CIs to all adults with severe-to-profound SNHL for improving speech understanding as well as overall communication and quality of life (QOL) (82).

Reasons for underuse include limited international guidelines, variable country-specific recommendations, low awareness and understanding of benefit, lack of access to hearing technology and/or hearing professionals, poor understanding of candidacy criteria, misconceptions about insurance coverage, and breakdowns in care pathways (86). Comprehensive guidelines for implementing HA and CI technology is an important first step toward improving utilization of hearing technology (89,90).

Clinics are treating a growing number of patients with greater amounts of residual hearing in the nonimplanted ear. These patients often benefit from a bimodal hearing configuration in which acoustic input from a HA on 1 ear is combined with electrical stimulation from a CI on the other ear. In 2010, Dorman and Gifford (91) reported that 60% of unilateral adult CI recipients had aidable residual hearing in the nonimplanted ear; more recently, Holder et al (92) reported this number had risen to 85% making bimodal candidates the most common patient profile seen by CI clinicians. Yet, there exists no current guidelines addressing bimodal fitting of hearing technology for the treatment of bilateral SNHL in adults. Further, recommendations on standard of care practices for bimodal fitting are lacking.

For unilateral CI recipients, the benefit derived from the addition of a HA on the contralateral ear is often referred to as “bimodal benefit.” Bimodal benefit can be significant even in cases where hearing thresholds may be deemed poor or “unaidable” (1,2,5). Patients often report that the CI provides saliant speech cues, while the contralateral acoustic signal provides the rich, natural sound quality to which they are more accustomed (18). Patients with tinnitus also report increased tinnitus suppression with the addition of a contralateral HA (14,93,94). Further, bimodal listening has been shown to provide objective benefits such as improved speech understanding in quiet and in noise (5,14,95,96), improved spatial hearing (14,60,97,98), and better music perception compared to the CI alone (19,99–105).

Although the addition of a contralateral HA can offer many advantages, it is important to remember that HAs and CIs should be considered part of a hearing healthcare continuum for all patients. Despite measurable hearing thresholds in the non-CI ear, some listeners receive no bimodal benefit with appropriately fitted amplification (6,106). In such cases, a second CI may yield greater benefit for speech recognition in quiet and noise (5,62,95,107) and spatial hearing (5,108–111). Regardless of the hearing loss configuration, hearing healthcare professionals should be consistently evaluating their patients’ outcomes and considering whether they may be better served by a different technological configuration such as 2 CIs instead of one.

There is an urgent need to address the lack of consistent guidelines for and awareness of the benefit of CI in combination with a contralateral HA for the treatment of bilateral SNHL in adults. The current guidelines are intended to review the literature and provide best practice recommendations for the evaluation and treatment of bilateral SNHL for those who may benefit from bimodal hearing configurations.

## BENEFITS OF BIMODAL HEARING

At present, approximately 80% of current adult CI recipients utilize a bimodal hearing configuration combining a unilateral CI with a contralateral HA (92). Furthermore, up to 85% of adults reporting for preoperative CI evaluation have aidable acoustic hearing, even if only in the low-frequency range (92). Researchers have repeatedly demonstrated significant bimodal benefit even for individuals for whom aided acoustic hearing alone offers little-to-no speech understanding (1,2,5). Bimodal benefit is observed for speech understanding, music perception and appreciation, and various aspects of spatial hearing.

Acoustic hearing offers access to both redundant information via binaural summation as well as unique or complementary information that is not well transmitted by modern-day CI systems. Acoustic hearing from the nonimplanted ear provides bimodal listeners access to F0, often referred to as voice pitch, and temporal fine structure providing cues for place of articulation and suprasegmental or prosodic speech features such as stress, tone, and intonation—all of which can be highly informative for conveying emotion, word meaning, and relative importance of spoken words. Suprasegmentals are particularly critical for tonal languages, for which bimodal hearing has been shown to yield superior speech perceptual outcomes as compared to CI-alone listening (112–115).

Auditory access to low-frequency F0 and temporal fine structure drives bimodal benefit; however, it is still unclear which of the following perceptual mechanisms is responsible for said benefits: 1) bimodal integration and 2) source segregation and/or glimpsing. “Bimodal integration” of acoustic and electric cues can occur for cues that similar across ears, or bilateral/bimodal redundancy (116,117) or the cues can be complementary (118). “Source segregation” is achieved when a listener can distinguish target talker F0 from the distracting talkers, thereby allowing the listener to segregate the source from the competing background. Bimodal listeners have demonstrated benefit from source segregation in various SIN environments, arising primarily from F0 access in the nonimplanted ear (7,9,19,96,119). Finally, bimodal listeners have consistently demonstrated benefits from “glimpsing” for which the bimodal listener utilizes acoustic hearing cues for voicing, manner, and fine structure to “glimpse” the target speech during spectrotemporal dips in competing backgrounds (15,120,121). Although we may not fully understand which auditory mechanisms are responsible for bimodal benefit—or if all are contributing differently to benefit in different listening scenarios—there is no doubt that combining a CI with contralateral HA affords significant hearing benefits for various perceptual tasks as outlined below.

### Bimodal Benefit for Speech Understanding

Bimodal hearing offers significant benefits as compared to unilateral CI alone for speech recognition in quiet and noise for adults (1–3,5–8) and children (9–13). However, this guideline will focus on adult CI recipients and associated bimodal benefit. Several studies have reported bimodal outcomes for clinical measures of speech understanding in adult CI recipients using both between- and within-subjects, repeated-measures designs. In within-subjects designs, researchers have consistently shown that bimodal hearing yields significantly higher outcomes as compared to the CI-alone condition for speech recognition in quiet (1,3,6,122) and colocated noise, for both steady-state noise (6) and competing talker backgrounds (1,3,5). Similarly, using between-subjects designs, studies have consistently demonstrated superior bimodal speech recognition outcomes as compared to individuals with unilateral CI (4,123). Mean benefit one might expect from adding an appropriately fitted and verified HA to unilateral CI listening is approximately 10- to 20-percentage points for speech recognition in quiet (1–3,5,6,14–16) and 10- to over 30-percentage points for both speech recognition in colocated noise (1–3,5,6,14,15,17) and in spatially separated noise (5,14).

### Bimodal Benefit for Music Perception and Appreciation

In addition to the bimodal benefit consistently afforded for speech understanding, bimodal hearing provides significantly better musical sound quality and music perception abilities over CI-alone listening, including benefits for chord, melody, and melodic contour recognition (99–101,103–105). More recently, research has shown significant bimodal benefits—over CI-alone listening—for music emotion recognition (20) and musical sound quality ratings across all musical genres (19). Thus, one may find that even in the absence of considerable bimodal benefit for speech understanding, the perceptual and sound qualitative benefits obtained from the contralateral HA would still be clinically and functionally significant.

### Bimodal Benefit for Environmental Sound Recognition and Overall Sound Quality

Despite the importance of speech and music stimuli in our everyday lives, there are environmental auditory stimuli that can provide us with critical information for our safety and overall QOL. Particularly important environmental sounds include vehicular noises, domesticated animal sounds (eg, barking, growling), phone alerts/rings, safety alarms (eg, smoke, carbon monoxide, low battery warnings), and various nature sounds arising from birds, insects, rain, and thunder. A recent study reported that adult bimodal listeners outperformed groups of both unilateral and bilateral CI users on tasks of environment sound recognition (124). Thus, there is emerging evidence that bimodal hearing is advantageous beyond the most widely used tasks of speech and music perception.

In addition to various tasks of auditory perception described here, there are also numerous published reports demonstrating the superiority of bimodal hearing over CI-alone listening for various subjective and qualitative aspects of sound. For example, bimodal hearing offers significant qualitative benefits resulting in a more natural, full, and pleasant sound quality for various dimensions of speech and music (16,18–21). Other studies have shown that listeners rate bimodal speech recognition to be significantly less difficult and less effortful as compared to CI-alone listening (16,22). Unilateral CI recipients also overwhelmingly report a preference for bimodal listening environments involving speech in quiet, noise, reverberation, and music (2).

### Patient Variables Influencing Bimodal Benefit

The success of bimodal hearing for auditory perception, sound quality, and listening effort has been demonstrated repeatedly over the past 2 decades. Although bimodal benefit is observed for the majority of adult CI users with acoustic hearing in the nonimplanted ear, the amount of observed benefit varies greatly across individuals (1–4). At present, we do not have strong predictor variables accounting for the variability observed in bimodal benefit. Unaided audiometric thresholds in the low-frequency range have either been shown to have no relationship with bimodal benefit (1,4) or are only weakly correlated with bimodal benefit (3,6,125). However, this weak relationship is largely driven by bimodal listeners with extreme audiometric thresholds such as those with completely normal hearing or audiometric thresholds ≥100 dB HL (3,6). In the event that an individual fails to obtain bimodal benefit from a well-fitted HA (see Bimodal Hearing Aid Fitting section), this same patient would still derive significant auditory benefit from unilateral CI (82) and hence would have nothing to lose from a trial with bimodal hearing.

### Summary of Benefits of Bimodal Hearing

1. Although bimodal benefit is highly variable per individual listener (1–4), it offers significant benefits on average as compared to unilateral CI alone for speech recognition in quiet and noise for adults (1–3,5–8) and children (9–13).
2. Mean expected bimodal benefit is approximately 10- to 20-percentage points for speech recognition in quiet (1–3,5,6,14–16) and 10- to over 30-percentage points for both speech recognition in colocated noise (1–3,5,6,14,15,17) and in spatially separated noise (5,14).
3. Bimodal hearing offers sound quality benefits (more natural, full, pleasant) for speech and music (16,18–21) and less effortful listening compared to CI alone (16,22).
4. Most CI users with hearing thresholds below 90 dB HL will derive benefit from a HA in the nonimplanted ear; however, if bimodal benefit is not demonstrated, a second CI should be considered (23).

## PREOPERATIVE CI EVALUATION AND SURGERY

Determining candidacy for CI is dependent upon many factors such as age, speech recognition, etiology of hearing loss, type of implant, and insurance/payer coverage. These factors can vary considerably between patients. This leads to wide variability in evaluation methods and protocols used to determine CI candidacy around the world. The complicated nature of establishing CI candidacy can result in uncertainty on when to refer patients for CI consult or when to transition HA users to CI. The following section will review essential parts of the CI candidacy evaluation and provide guidance on the appropriate time to refer patients for an evaluation. Although the focus of the evaluation is the audiologic assessment, medical, radiologic, and psycho-logical factors will also be reviewed, as they must be equally considered prior to determining candidacy.

### When Is the Appropriate Time to Refer for a CI Evaluation?

Evidence from the HA literature shows that when thresholds exceed 70 dB HL, amplification benefit is diminished (50,126–132) due to stimulation of cochlear dead regions (96,132,133), distortion at such high output levels (50,134–136), and/or negative effects of high input compression ratios (135,137–139). In such cases, listeners would likely be better served by a CI (24).

There is also evidence to show that patients with lesser degrees of hearing loss benefit from traditional CI (140–143). The most common hearing loss configuration in adults is high-frequency sloping hearing loss, and this mid-to-high frequency range contains critical information for discerning speech in background noise and perceiving place of articulation cues (eg, /f/ vs./s/). Even if low-frequency thresholds are in the moderate range, once the high-frequency thresholds reach the severe-to-profound range, Hogan and Turner (128) showed that the addition of aided audibility in this range was not useful. Patients with sloping hearing loss profiles will generally show poor speech understanding in quiet without visual or context cues, exhibit significant difficulty understanding speech in background noise, and often report that they can hear but not understand. While these patients may show slightly higher speech recognition with their HAs than the severe-to-profound group, several studies have shown that they would likely be better served by a CI (eg, [141,142,144]). Further, it is important to remember that the audiogram does not reflect that patient’s auditory function or perception, so the decision of whether or not to refer should not be based solely upon the audiogram (145).

Zwolan et al (24) recently provided a screening procedure for referring patients for CI evaluation. They found that using a “60/60 referral guideline” yielded a 96% detection rate for identification of adults who met traditional CI candidacy. The “60/60 guideline” suggests providers should refer adults with hearing loss for a CI evaluation when they present with ≥ 60 dB HL 3-frequency PTA and ≤ 60% unaided word recognition score in the better hearing ear (24). While these protocol guides serve as a starting point, referring providers should consider referring any consistent HA user whom is not adequately benefitting from appropriately fitted HAs: to a CI team for a formal CI evaluation. The referring provider is not obligated to determine candidacy, so no referral is a poor referral. CI candidacy evaluations that do not result in immediate implantation provide an opportunity for patient education and preparation for later implantation in cases of progressive hearing loss.

If the information necessary to assess the 60/60 referral guideline is unavailable, but the patient answers “no” to any 2 of the following questions, a referral may be necessary:

- Are you able to talk on the phone without visual cues (such as video or captions)?
- Are you able to understand television programs without closed captioning?
- Are you able to effectively engage in conversations at large group gatherings such as dinner parties?
- Do you feel that you obtain significant communication benefit from your current HAs?

### What Is Involved in a CI Candidacy Evaluation?

The typical CI evaluation starts with otoscopy and tympanometry to rule out outer or middle ear abnormalities. Then, it is recommended that the audiologist complete a standard audiometric evaluation including pure tone air and bone conduction thresholds to assess hearing sensitivity. Air conduction thresholds should include 125 Hz, as it can be an important marker of minimally traumatic surgical technique and/or low-frequency hearing preservation, which has been correlated with more favorable outcomes in the literature (146–148).

Following comprehensive audiometric testing, the patient’s current HA settings should be verified to ensure the HAs are optimized prior to completing aided testing. It is recommended that the HAs be verified using a probe microphone approach to ensure that the HAs are providing the appropriate gain prescribed by a validated prescriptive method (ie, NAL, DSL) (149). If the HAs are not matching targets, the HAs should either be reprogramed or stock HAs should be programmed and verified to target for preoperative aided testing. HA verification is essential to the CI candidacy evaluation process, yet evidence suggests that only 29%–50% of individuals referred for CI evaluation are wearing appropriately fitted HAs (89,92). This part of the candidacy evaluation is crucial to be sure that all nonsurgical options for improving the patient’s hearing are exhausted prior to recommending implantation.

It is important to recognize that patients with similar audiograms can have drastically different speech understanding abilities; thus, it is critical to consider a patient’s complete audiometric profile. The candidacy evaluation should include assessment of auditory only speech understanding performed with appropriately fitted and verified HAs. The patient should be instructed to listen to words, sentences, and sentences in background noise and repeat what they hear, guessing if necessary. Speech stimuli should be presented from a loudspeaker at 60 dB SPL (150), representing the typical loudness level of conversational speech. Presentation at higher levels (ie, 70 dB SPL) should be avoided because such levels are not vocally sustainable in realistic communicative environments (151) and can artificially inflate speech recognition scores (140). Unless otherwise not possible, speech stimuli should always be presented using recorded stimuli, as speech recognition presented using monitored live voice has been found to be unreliable and a poor predicter of CI candidacy (152,153). Speech materials should be calibrated using a sound level meter to ensure accurate presentation levels. At minimum, speech stimuli should be presented to each ear individually and ideally in the bilateral HA condition. In addition to assessing speech recognition in quiet, it is recommended that speech also be assessed in the presence of background noise using a SNR of +10 or +5 dB (142,154–156). Testing in background noise is important because it is often the patient’s greatest complaint (154), and it simulates common real-world communication situations (155,156).

Another important component of the CI candidacy evaluation is the administration of questionnaires. While the use of questionnaires is not standardized, it is widely agreed upon that questionnaires are a valuable tool to assess patient perceived hearing difficulty, QOL, and expectations for CI. Further, such questionnaires may serve as useful tools to validate the efficacy of CI and track outcomes over time. Commonly used validated questionnaires include the Speech Spatial and Qualities questionnaire (157), Abbreviated Profile of Hearing Aid Benefit (150,155,158), Cochlear Implant Quality of Life (CIQOL) Questionnaire (159), and the Nijmegen Cochlear Implant Questionnaire (160). While a validated expectations tool does not yet exist, in addition to these questionnaires, an expectations questionnaire is recommended to document and counsel on appropriate expectations prior to implantation. In some cases, a referral to a psychologist may be warranted to establish appropriate expectations, ensure motivation for rehabilitation, or help patients cope with their hearing loss.

Specific recommendations for candidacy evaluation protocols can be found in the Minimum Speech Test Battery (MSTB) (23) with further explanation in “Cochlear Implant Patient Assessment: Evaluation of Candidacy, Performance, and Outcomes” (161), and an example of how these protocols are implemented in a major academic medical center in the United States can be found in Holder et al (92). It should be noted that the assessments contained within the MSTB represent the current standard of care for CI candidacy evaluations in the United States.

In addition to the audiometric assessment, the patient should have a consult with an otolaryngologist or ENT surgeon. The surgeon should complete a thorough evaluation of the ears and order and review appropriate radiographic images (computed tomography and/or MRI scans) to assess anatomy of the cochlea, vestibule, and internal auditory canal. Additionally, pneumococcal vaccines are recommended to be administered as per the centers for disease control and prevention guidelines (https://www.cdc.gov/vaccines/vpd/mening/public/dis-cochle-ar-faq-gen.html) to prevent certain types of meningitis, which occurs with increased frequency in patients with CIs. Following these appointments, a decision regarding candidacy should be made with input from all members of the CI team.

### What Should the Patient Expect During and After Surgery?

Most CI surgery is performed in an outpatient setting with patients going home the same day. For patients with medical problems, a preoperative assessment by their family doctor or the anesthesia team may be recommended to minimize risk. Informed consent should be obtained by going over the risks and benefits of surgery with the vast majority of risks occurring exceedingly infrequently. The risk most concerning to patients is facial nerve injury resulting in a drooping face on the side of implantation. Fortunately, this complication is exceedingly rare, and if encountered immediately following surgery, it is usually associated with complete recovery of facial function over time (162,163).

Surgery should be performed by an appropriately trained surgeon. The procedure is usually performed under general anesthesia and typically takes approximately 1 hour of operative time with a total time of approximately 3 hours including induction and recovery from general anesthesia. Postoperative recovery typically takes a couple days during which time pain is controlled with a course of surgeon prescribed narcotic and/or non-narcotic pain meds (eg, acetaminophen). Several side effects have been noted following CI surgery. Some patients have postoperative disequilibrium/dizziness, which typically resolves over the ensuing days to weeks (164). Long-term disequilibrium does occasionally occur and may be worse in elderly patients (165). Almost every patient complains of ear numbness secondary to the postauricular incision. Fortunately, this slowly improves over several months. Additionally, about 1 in 5 patients have long-term taste disturbance on the ipsilateral tongue due to irritation and/or injury of the chorda tympani branch of the facial nerve (166). Clinically significant postoperative infections requiring explantation and reimplantation occur in less than 1% of cases (167). Long-term device failure requiring reimplantation occurs with a lifetime incidence of approximately 4%–5% (168,169) with updated data reported from each company annually. While there are case studies of immediate postoperative activation, most centers and patients prefer to wait for activation until the postauricular incision has healed and soft tissue swelling has resolved, which takes approximately 2 weeks.

As far as surgical procedures go, CI surgery is generally one of the simpler surgeries that a well-trained otologist completes. Of course, any surgery must be approached with informed consent with the patient having complete trust in their surgeon. With a well-informed and highly experienced team, the CI surgery is but a brief step in the process toward better hearing.

### Summary of Preoperative CI Evaluation and Surgery

1. Providers should refer adults with hearing loss for a CI evaluation when they present with ≥ 60 dB HL 3-frequency PTA and ≤ 60% unaided word recognition score in the better hearing ear (24).
2. CI candidacy evaluation should consist of standard audiometric testing, aided speech recognition testing using an appropriately fitted and verified HA, questionnaires, ENT consult, radiologic imaging, and other referrals necessary for a specific patient (psychology, anesthesiology, speech-language pathology, etc.) (25).
3. CI surgery is typically completed in an outpatient setting with quick recovery time and minimal complications.

## Postoperative CI + HA fitting and care

### Postoperative CI Fitting and Assessment

#### Expectations Regarding CI Activation

The CI activation appointment can be overwhelming, exciting, and scary for the patient and their loved ones. It is often helpful if the CI patient brings family members or friends to the initial activation session as a means of emotional support. While clinicians typically counsel patients regarding realistic expectations prior to the initial activation appointment, frequently, the CI recipient and their family arrive to the session with unrealistic expectations. Many patients are disappointed when the CI device is turned on due to their inability to understand speech immediately following activation. However, patients should be encouraged that speech quality and understanding will improve over the first few months following initial activation (26). Although improved speech understanding and sound quality takes time to develop, typically, the patient will be able to hear sounds when the device is activated, if the internal CI device is appropriately placed in the cochlea and the patient has a functioning auditory nerve. Due to the difference of frequency allocation of the CI versus the normal auditory system (170) and due to the patient’s hearing history, speech recognition and sound quality outcomes with the CI are variable. Some adult CI users will not understand speech at the time of the initial activation and will simply hear sounds (ie, beeps, bells, static) when the audiologist or family member is talking to them. Other patients are capable of understanding words and short sentences, but they report speech sounds abnormal (ie, duck quacking, Minnie Mouse, Darth Vader). The patient’s brain will require days, weeks, or months to adapt to the electrical signal produced by the CI before speech understanding improves. On average, speech understanding continues to improve over “the first year of CI use” (26).

The initial activation session will last anywhere from 1 to 2 hours depending on the clinic. Prior to the patient arriving for the appointment, the audiologist will review the surgical report and the postoperative imaging, if available, to confirm device placement in the cochlea. The audiologist will begin with otoscopic inspection and visual assessment of the incision site to assure it is safe to activate the device. Next, the magnet strength will be verified by attaching the coil or headpiece over the internal device site. If the magnet is too weak, the coil or headpiece will fall off and interrupt sound transmission. If the magnet is too strong, skin necrosis can occur and, while rare, subsequent need for reimplantation may be necessary if left untreated. After determining the appropriate magnet strength, the audiologist will attach the CI speech processor to the computer and begin the CI activation process.

#### Determination of Lower Stimulation CI Programming Levels

Appropriate programming of the threshold (T) level for CI recipients is necessary to provide audibility for soft sounds. Advanced Bionics (Valencia, CA), Cochlear (NSW, Australia), Med-El (Innsbruck, Austria), and Oticon Medical (Vallauris, France) each describe their recommendation for setting the T level differently. Advanced Bionics recommends setting the T level at the lowest stimulation level the patient hears 50% of the time. Cochlear recommends setting the T level at the lowest stimulation the patient hears 100% of the time. Med-El recommends setting the T level just below the lowest stimulation level the patient can hear (ie, T level should not be detectable). Oticon Medical recommends setting stimulating T levels in groups of 3–5 electrodes and setting them at a level that is reported as “very soft”; if T levels are measured individually, they recommend setting the T at a level that is barely audible.

There are several methods available to program the T levels needed for electrical stimulation of the CI device. The patient may be asked to listen to soft sounds and indicate when they hear the sound similar to a hearing test, or the patient will be asked to count the number of beeps heard. T levels are set accordingly. It is difficult for patients with tinnitus (171,172) and/or long durations of deafness to correctly set T levels behaviorally at the initial activation session, and this measurement is often deferred to a later programming session. Recent programming recommendations for Med-El and Advanced Bionics suggest setting the T levels to 0% or 10% of the upper stimulation levels will allow for sufficient access to soft sounds and will reduce the time needed for programming (173). If T levels are set too high, or too loud, it can result in perception of circuit noise and unnecessary compression of the electric dynamic range (174). Conversely, if T levels are set too low, patients will not have adequate access to soft levels of speech, which will hinder their overall success with the CI (27). The clinician should verify that T levels are appropriately set by conducting aided detection testing in the sound field using warble tones. Aided detection thresholds should be obtained in the 20–30 dB HL range for 250 to 6000 Hz to ensure appropriate access to speech sounds (27). If detection thresholds are higher or lower than this range, adjustment to the T levels is warranted.

#### Determination of Upper Stimulation CI Programming Levels

Appropriate determination of the upper stimulation levels (M, MCL, or C levels depending on the CI company) for adult CI recipients is another important aspect of CI programming (31,37,175–177). If upper stimulation levels are set too high the overall stimulation may cause discomfort to the patient and in worst cases can cause facial nerve stimulation. If upper stimulation levels are set too low, speech understanding can be compromised.

There are various methods available to set upper stimulation levels for adult CI patients, and they are divided into 2 categories: behavioral and objective measures. Setting upper stimulation levels via behavioral measures forces the audiologist to rely on patient report. The audiologist can use loudness scaling, which means the patient listens to ascending levels of stimulation on various electrode channels and reports on the loudness of the beeping sound, or the audiologist will simply turn on the CI stimulation in live speech mode and globally increase all sounds to a patient-reported comfortable level. Advanced Bionics, Cochlear, Med-El, and Oticon Medical each describe their recommendation for setting the upper stimulation levels differently. Advanced Bionics recommends setting at the “most comfortable level.” Cochlear recommends setting at “loud, but comfortable.” Med-El recommends setting at the “maximum comfort level.” Oticon Medical recommends “medium comfort” if stimulating a single electrode, “comfortably loud” if stimulating 2–3 electrodes, and just below “maximum comfort” if stimulating 5 electrodes during measurement.

In addition to loudness scaling, loudness balancing and sweeping have been shown to be a critical components of CI programming (178). During loudness balancing, the audiologist will ask the patient to listen to 2 neighboring electrode channels and determine if they sound similar in loudness. Upper stimulation level limits will be altered slightly based on patient report. Loudness balancing is performed across the entire electrode array to assure all channels are equal in volume. Sweeping is a task used to stimulate electrode in a sequential manner to assure none of the electrodes cause discomfort or result in abnormal sound quality.

Behavioral measures such as loudness scaling and balancing are prone to error because loudness is highly variable in individuals with hearing loss especially those with longer duration of deafness (37–40). Objective measures such as eCAP and eSRT do not require patient report and are the most commonly used objective measures for estimating upper stimulation levels. Automatic and manual eCAP testing is available in the CI company software and is useful for confirming electrode function and neural response, as well as monitoring change in device function over time. However, eCAPs are poor predictors of upper (and lower) stimulation levels and have shown cross-electrode and cross-subject variability (29,41–48). As a result, it is recommended that clinicians do not rely solely on eCAP measurements when setting CI stimulation levels.

A less commonly used but more accurate objective approach to programming upper stimulation levels involves the use of eSRTs, which provide an objective correlate to a stimulation level and overall upper stimulation level profile shown to be perceived as “loud but comfortable” on average (28–36). MAPs using eSRTs to set upper stimulation levels have shown equal (31,34) or better (177,179) speech recognition results compared to behavioral-based (loudness scaling) maps. Further, eSRT-based MAPs have been shown to result in equal loudness across the electrode array, and patients tend to prefer eSRT-based MAPs over behavioral MAPs (39). Anecdotally, eSRTs can be especially useful for setting the upper stimulation levels of high-frequency electrodes as adult recipients are prone to reporting that stimulation on these electrodes is too loud when in actuality, it is the pitch to which they are averse.

#### Expected Postoperative Follow-up Schedule(s)

Clinic recommendations for the CI postoperative activation and programming schedule vary across centers. The suggested timing for initial activation is dependent on the clinic, how the patient has recovered from surgery and if postoperative surgical complications are noted. The initial activation session occurs an average of 28 days postsurgery, according to a recent survey of CI centers (180). While there is no consistency in the literature regarding specific recommendations for a postoperative follow-up schedule for adult unilateral CI recipients, it has been suggested that patients should be programmed more frequently in the first few months following initial activation and less frequently thereafter (181–185). In the first year after surgery, adult unilateral CI patients are generally seen for 4–6 programming sessions: initial activation, 1–2 weeks post-activation, 1, 3, 6, 12 months post-activation (23,49). CI stimulation levels stabilize within the first year after activation, however, patients usually return annually or every 2 years for the remainder of their lifetime (181,182). Additional programming sessions are scheduled if the patient reports a change in hearing, if equipment is malfunctioning, and/or if new equipment becomes available.

#### Postoperative CI Assessment Best Practices

It is important for the CI clinician to monitor the patient’s access to soft sounds at each programming session with a frequency-specific CI sound field aided audiogram using warble tone threshold detection. An optimized CI audiogram has subjective thresholds in the 20–30 dB HL range for 250 to 6000 Hz (27). If the CI audiogram is outside of the recommended hearing range, stimulation levels should be adjusted. It is recommended that patients undergo CI speech perception testing at regular intervals post-activation (3, 6, and 12 months) to track outcomes longitudinally (186). The recommended MSTB (23) includes Consonant-Nucleus-Consonant words (187), AzBio sentences (in quiet and noise) (188), and Bamford-Kowal-Bamford Speech-in-Noise (189). The CI patient should sit in a calibrated sound booth with speech and noise presented at 0° to azimuth at a presentation level of 60 dB SPL for quiet conditions and 65 dB SPL for noisy conditions (23). Test conditions should include each CI alone and the bimodal condition if applicable. While routine assessment of speech outcomes for adult CI users is important to track progress with the CI, it is also used to identify potential problems with the CI. A clinically significant decline in speech understanding can be a red flag for a problematic internal CI device (190).

#### Summary of Postoperative CI Fitting and Assessment

1. Realistic expectations for activation and postoperative improvement are important. Initial sound quality with the CI is variable but will typically improve over the first few months following initial activation with continued improvement in speech understanding over the first year of CI use (26).
2. Lower stimulation levels should be programmed according to manufacturer recommendations and verified using aided detection testing. Aided thresholds should be in the 20–30 dB HL range for 250 to 6000 Hz using FM warble tones to ensure appropriate access to speech sounds (27).
3. Upper stimulation levels should be optimized using eSRTs to ensure that they are set appropriately (28–36) as behavioral measures such as loudness scaling are variable (37–40), and eCAPs are poor predictors of stimulation levels (29,41–48).
4. CI patients should return for follow-up to fine tune the CI programming and assess outcomes of the implanted device (23,25). Follow-up schedules vary but typically include 4–6 sessions in the first year of implantation (23,49).

## Bimodal Hearing Aid Fitting

While fitting the CI and HA separately have been well-described in the literature, evidence on HA fitting procedures for bimodal CI users is lacking. Results from multiple international and US surveys revealed that most clinicians advise CI recipients to wear a contralateral HA if indicated, yet no dedicated bimodal HA fitting protocols were clinically applied (191–193). Nevertheless, several CI manufacturers provide specific HA fitting recommendations for bimodal CI users (194–196) based on current, but scarce, evidence and clinical practice. One manufacturer developed and marketed a dedicated bimodal fitting formula (197), yet varied results in bimodal auditory performance have been reported (51,52,56,57,198). Another manufacturer promotes reducing the device delay mismatch between HA and CI to improve localization abilities of bimodal users (199) with varied results reported in the literature. Regardless, the vast majority of CI audiologists recommend use of the CI manufacturer’s partner HA when available (191), likely due to multiple factors including cost, clinician comfort, ease of programming, and patient benefit of bimodal streaming and Bluetooth compatibility.

A recent systematic review summarized the findings of the peer-reviewed literature on bimodal HA fitting (51). This review, along with more recent published literature are categorized into 5 topics for bimodal fitting considerations:

1. Frequency response of the HA
2. HA fitting formula
3. Use of frequency lowering technology
4. Synchronization of AGC between HA and CI
5. Interaural loudness balancing

### Frequency Response of the HA

In a systematic review from 2018 (51), the majority of studies on bimodal HA fitting included the effect of HA frequency response on bimodal performance in the design of the study (12,51,53,59–61,200–204). However, only 3 studies (12,200,202) compared relevant outcome measures for different setting of the frequency response, without varying other fitting factors. A couple of recent studies analyzing bimodal performance as a function of the HA bandwidth found that bimodal patients achieve best audibility with the widest bandwidth (19,205). In general, wideband amplification resulted in equal or better performance compared with band-limited amplification (19,51,205). This suggests one should only band limit the response in special occasions, such as feedback problems of the HA, user complaints about poor sound quality, or the presence of cochlear dead regions (206). In cases where dead regions are a concern, clinicians could implement the threshold equalizing noise (TEN) test (207,208) to assess for dead regions (TEN test can be obtained here: https://www.psychol.cam.ac.uk/hearing/cds-for-diagnosis-of-dead-regions-in-the-cochlea-2013-ten-hl-and-ten-er3). If dead regions are present, the bimodal HA could be programmed using either full or restricted bandwidths (209).

### HA Fitting Formula

The studies included in this section compared different fitting formulas (51–55,198) or the application of shifts or tilts to a predefined frequency response (53,59–61). Results suggest a prescribed fitting based on NAL or a similar prescription rule is a good starting point in bimodal HA fitting and may even provide a (near)-optimal solution for the majority of bimodal users (51–55). One study found improved speech perception and bimodal benefit with DSL v5.0 as compared to NAL-NL 2 (55). Subjective preference was noted for company-specific proprietary fitting formulas in 1 study using Advanced Bionics’ Adaptive Phonak Digital Bimodal Fitting Formula (APDB) (197) and 3 studies using ReSound’s Audiogram + fitting formulas (52,56,198). However, variable results were found when comparing NAL to APDB for SIN test conditions (52,56), and no significant improvement was noted for speech in quiet using APDB or Audiogram+ (52,54,198). Individual fine tuning may be helpful for a subgroup of bimodal users, although the resulting effect on auditory performance remains unclear. More comparative HA fitting studies for bimodal CI users are needed to determine which prescription rule provides optimal bimodal performance for which patient.

### Frequency Lowering Technology

Six studies examined the effect of frequency lowering technology on bimodal auditory performance (12,210–214). No differences were found in bimodal auditory performance for fitting strategies with and without frequency lowering, except for the study by Perreau et al (213). In HA patients, frequency compression or transposition has shown to have the largest effect in patients with precipitously sloping hearing losses in the high frequencies (215,216). In the included studies on this topic in bimodal CI users, the type of hearing loss was heterogeneous between subjects (steep sloping hearing losses as well as relatively flat hearing losses were included). It is possible that, when selecting subjects with relatively good low-frequency hearing and precipitously sloping high-frequency hearing loss, more benefit can be found. Future research on this topic should also focus on the effect of frequency compression for these hearing losses. For now, current evidence suggests that frequency lowering or transposition is not beneficial for bimodal CI users (51).

### Synchronization of AGC Between HA and CI

Dynamic compression is a possible relevant factor in HA fitting for bimodal CI users that may be easily overlooked. The hypothesis is that matched AGC helps to equalize loudness between HA and CI when the devices are in compression, which is favorable to binaural processing. However, the effects on auditory performance of synchronizing the dynamic compression between HA and CI are varied. A recent study reported improved spatial hearing abilities with synchronized AGC (58) but did not assess speech recognition outcomes. Two other studies (56,57) found a significant bimodal benefit for the AGC-matched HA as compared to the standard AGC setting for SIN test conditions, but no significant difference was found for speech in quiet. Conversely, in a study by Vroegop et al (52), no difference in auditory performance was found when using the same AGC-matching as used in the study of Veugen et al (57). It is evident that more data are needed to provide clarity on this topic.

### Interaural Loudness Balancing

Two studies (54,57) compared 2 different loudness-balancing methods. They did not find any difference in performance between broadband and 3-band loudness balancing. Other studies (51–53,59–61) showed that loudness balancing only had a moderate effect on the provided gain. However, individual differences were quite large. More research is needed to provide insight for which patients balancing is needed and maybe provide additional bimodal benefit.

### Clinical Implications

The existing literature reveals that although bimodal benefit was found in many of the reviewed studies, no clear evidence exists on best HA fitting protocols for optimal bimodal performance, with the exception of real-ear measurement utilization, which was commonly noted to provide bimodal and subjective benefit (209). As the number of CI candidates with residual hearing continues to rise, the need for bimodal management best practices is eminent. While use of HA technology on the contralateral ear is generally recommended for CI users with aidable hearing (191,217), it can also prove beneficial even for those with significant contralateral hearing loss (122) who are unable to access bilateral CI as a treatment option. With the increasing number of bimodal CI users globally, CI clinicians should upgrade their knowledge on HA fittings and incorporate bimodal management of the CI and HA into their clinical practice. Aided speech recognition testing is always recommended to ensure best outcomes for bimodal listeners. Further research is clearly warranted in HA fittings for optimal bimodal performance.

### Summary of Bimodal HA Fitting

1. Real-ear verification of the aided response should be the standard of care when fitting the HA as adequate audibility is essential for HA benefit (50). No clear evidence was found on how certain choices in HA fitting formulas contribute to optimal bimodal performance. A standard fitting formula for severe hearing loss for which the target HA aided response is known, like NAL-NL 2 or DSL-5, is recommended (51–55).
2. Current evidence suggests that frequency lowering is not beneficial for bimodal CI users (51).
3. Synchronization of AGC between HA and CI is possibly beneficial (56–58); however, more research is needed to this topic. Currently, the matched-AGC approach is only clinically available with Advanced Bionics’ bimodal system. While CI clinicians can certainly alter the AGC in the HA software for other devices, there is no research to support this approach at present.
4. The additional value of interaural loudness balancing between HA and CI is not clear as it typically does not result in large deviations from the prescribed gain by the initial fitting formula (51–53,59–61).

## EXPECTATIONS AND OUTCOMES OF UNILATERAL CI USERS WHEN WEARING A CONTRALATERAL ROUTING OF SIGNAL DEVICE

Globally, the access to “bilateral” CI, for individuals with bilateral severe-profound SNHL, is limited (218). This section is designed to provide evidence-based recommendations for the use of CROS technology in unilateral CI users when bilateral implantation is not possible. Although the CROS technology is no longer available with the most recent CI processor release, the following information remains applicable to previous generations.

Binaural hearing provides access to acoustic cues required for everyday listening tasks such as perception of speech in background noise and localization of sounds in space. In the absence of binaural hearing, these cues are largely disrupted leading to the inability to effectively segregate auditory streams (219). A large body of evidence supports that bilateral CI can improve these processes in bilaterally deafened individuals (63), while these processes remain grossly impaired unilateral implantation (220–222). Further, the psychosocial consequences resulting from the communication impairments associated with unilateral hearing are often deleterious (157,223–225). At present, the vast majority of bilaterally deafened individuals are limited to a single unilateral CI (218). This is unfortunate as unilateral CI listeners are not able to extract the binaural difference cues required to locate sounds and segregate speech from interfering signals such as noise. Principally, in a monaural listening condition, all sounds arrive to the monaural hearing ear at the same time and intensity (219). Psychophysical evidence (226) indicates that acoustic cues arising from the acoustic head-shadow may be adapted and processed monaurally to provide improved performance for some listening tasks such as speech perception in noise. There is well-established evidence for the benefits of CROS in alleviating the negative consequences of the acoustic head-shadow in a monaural listening condition (227–229). Specifically, CROS allows monaural listeners to regain access to sounds arriving at the deaf ear by using a microphone and transmitter to reroute the signal to a receiver worn in the better hearing ear. The effects of improved speech perception with rerouting are most prominent when speech is directed to the CROS aided ear in spatially separated noise (227–230), although improved performance may also be observed in diffuse noise (64,71). It is important to note that performance outcomes with CROS technology in traditional monaural listeners (ie, those with single-sided deafness) (228) may not be directly translated to unilateral CI users (72). Specifically, a healthy cochlea has approximately 3000 frequency-specific channels compared to the limited channels provide by a CI, which is subject to spread of excitation and channel interaction (231). Monaural processing differences between the normal cochlea and the CI are most notably observed in the processing of speech, and therefore may play a role in hearing outcomes and perceived benefit of CROS in unilateral CI users (72). There are, however, fundamental similarities of CROS that will be used to generate the following recommendations.

### Recommendations

Although binaural cues are not well represented in bilateral CI or bimodal patients, bilateral stimulation provides direct and independent stimulation of each ear, potentially allowing for some binaural processing (65,66,68,69,232). Evidence has demonstrated improved speech perception in noise and localization performance in bilateral CI users (108,220,221,233,234), indicating that interaural level differences cues can be realized to some degree in these listeners (62,67,108). For these reasons, where possible, bilateral stimulation through CI is recommended for individuals with bilateral severe-to-profound SNHL unless otherwise contraindicated.

CROS technology should be reserved for use in unilateral CI patients with severe-to-profound hearing loss in the contralateral ear, unaidable by means of a traditional HA, and where access to a second CI is not possible (ie, extreme durations of deafness, insurance denial, anatomical contraindications). In some cases, CROS may be utilized in unilateral CI patients with residual hearing in the contralateral ear where traditional amplification has proved ineffective and access to a second CI is not possible. This may be observed in patients with longstanding hearing impairment where the nonimplanted ear has gone without stimulation, leading to patient rejection of the contralateral HA or complaints of binaural interference, thereby reducing benefit obtained by the unilateral CI alone. In such cases, CROS may be applied to provide the unilateral CI listener with access to sound from the nonimplanted ear in a way that provides a clear(er) signal to the better performing auditory system (70).

The benefits for bilateral CI can be largely attributed to the head shadow, allowing listeners to attend to the ear with the more favorable SNR when listening in spatially separated speech and noise (220,221,232). In unilateral hearing conditions, the head shadow negatively affects listening, particularly when noise is masking the better hearing ear and the head creates an acoustic barrier for targets directed at the deaf ear. CROS can also overcome this by rerouting the target signal to the better hearing ear and has been shown to improve the SNR for deaf ear listening on the order of 7–9 dB (227,228). Localization of sounds is not improved through rerouting of signal (72,227,228) but may be improved through bilateral CI (108,220,221,233,234).

Unilateral CI listeners are at a significant disadvantage for understanding speech directed to the nonimplanted ear in competing noise (64,70,71). Dorman et al (64) demonstrated a decrease of approximately 28% in mean word understanding for speech directed to the nonimplanted ear compared to the unilateral CI ear in competing noise. Similarly, the increase in SNR required to overcome the negative effects of the acoustic head-shadow for talkers located at the nonimplanted ear is inordinately high in unilateral CI users (70–72) compared to traditional unilateral listeners (228).

Primary benefit of CI + CROS is realized when speech is directed at the nonimplanted ear in competing noise (64,70,72,73). Smaller yet significant improvements have also been noted for speech in front of the listener (64,70,72). The CROS device is expected to disrupt speech understanding when the unilateral CI has the more favorable SNR (71,74,235). However, the observed negative affect is marginal (64,71,72,75) in comparison to the benefit gained when the more favorable SNR is at the CROS ear. CROS technology also appears to reduce the asymmetries in hearing performance that occur as a function of talker location for unilateral CI listeners (64,72).

Success of CROS should be determined through comprehensive behavioral and subjective outcome assessment. There is limited evidence on the long-term adoption and acceptance of CI + CROS. Mosnier et al (70) found high rates of self-perceived satisfaction in a sample of 8 CI + CROS users, and this was maintained over a period of 12 months. Additional studies of long-term benefit of CI + CROS are needed. Behavioral outcomes assessment should include tests of head-shadow to best determine the hearing deficits experienced by the unilateral CI listener as it relates to the potential benefits of applying CROS technology (64,70,72,73).

### Summary of Evidence for Selecting a CROS Device

1. Bilateral (62–67) or bimodal (68–70) stimulation should be prioritized unless otherwise contraindicated.
2. The greatest deficit for speech perception in noise in unilateral CI users is observed when the CI is masked by competing signals and the target is directed to the nonimplanted ear (62,64,71–73).
3. CROS is effective in improving the SNR at the deaf ear in unilateral CI users (62,64,71,72,74) and may improve hearing outcomes for targets in front of the unilateral CI listener (62,64,71,72,75).
4. Negative effects of CROS can be observed when competing signals (ie, noise) is transferred to the unilateral CI, although this is small in degree (64,71,72,75).
5. CI + CROS provides comparable benefit for lifting of head-shadow to bilateral CIs (64); however, localization is not improved by CI + CROS.
6. The most reliable method of validation CROS benefit is utilizing behavioral tests of head-shadow using measures relative to threshold to detect changes. Fixed SIN measures of < +5 dB SNR may be too challenging for unilateral CI users and may underestimate CI + CROS benefit (64,72,75).

## CI + HA FOR TINNITUS SUPPRESSION

Tinnitus is defined as the perception of sound(s) in the absence of an external stimulus. Patients with normal hearing and/or varying degrees of bilateral or unilateral hearing loss (mild to profound) have reported debilitating tinnitus. It is one of the most common otological complaints affecting approximately 4% of the general population in the United States (236–244) and 4.6% to 30% of other populations (236–244).

Tinnitus is often reported to be a bigger perceived problem than hearing loss. While various tinnitus suppression treatments have been developed, no pharmaceutical or surgical tinnitus treatment has been approved by the US Food and Drug Administration. Although most adults who suffer from tinnitus seek less invasive options (eg, prescription pill and/or counseling) to reduce tinnitus, many are willing to undergo CI to alleviate the effects of their tinnitus (245).

### Bilateral Hearing Loss and Use of CI for Tinnitus Suppression

William House (246) was the first to report on tinnitus suppression after CI. Several recent studies have provided additional support of Dr House’s findings (247–250) including a recent meta-analysis of 27 studies, which concluded that CI patients report significant improvement in tinnitus following implantation (251). Successful use of a CI to treat patients with tinnitus indicates electrical stimulation of the auditory nerve might be able to reverse the reorganization associated with peripheral deafferentation that causes tinnitus, thus, reversing plastic changes that may have caused the tinnitus. Also, the increase in activation of the auditory nerve may provide inhibitory influence on the cells in the auditory nervous system, which may play a role in its effect on tinnitus. Enhanced attentiveness to environmental sounds could also contribute to the observed suppression of tinnitus in patients with a CI (252).

### CI for Asymmetric or Unilateral Hearing Loss and Incapacitating Tinnitus

With advancements in technology and recognized benefits of improved speech understanding in quiet and noise with electrical stimulation (253–256), the potential to expand implant criteria has recently begun to include the application of CIs to subjects with asymmetrical hearing loss and unilateral hearing loss with severe tinnitus. Previous studies have indicated tinnitus in unilateral hearing loss can be severe and refractory to treatment (257). Many studies have shown that use of CI in unilateral hearing loss can help relieve tinnitus experienced by patients (258–262). For some bimodal listeners, it is possible to integrate acoustic hearing with CI stimulation to reduce troublesome tinnitus (14). Additionally, the development of modified shorter electrode arrays used to preserve low-frequency acoustic hearing allow some benefits for those patients who experience tinnitus (263).

### CI Fitting Options for Tinnitus Relief

Most CI users experience relief from tinnitus with use of their CI sound processor and typically do not require special CI programming parameters. However, it can be difficult for audiologists to accurately set stimulation levels for CI users with tinnitus. Tinnitus is easily mistaken for CI stimulation during programming, creating confusion for the patient when behaviorally measuring threshold levels (171,172). Difficulty in programming upper stimulation levels has been noted as well due to the patient’s intolerance for levels that can elicit tinnitus (264). While there is no unique protocol for programming CI recipients with tinnitus, clinicians should be able to employ alternative programming strategies when necessary to appropriately program the CI. Recipients should be counseled to use the CI for all waking hours to alleviate tinnitus perception, but they should also be educated that when the processor is not worn, the tinnitus may be noticeable.

### Sound Therapy for Tinnitus Relief

Many patients with mild to severe hearing loss report some relief from their tinnitus when using HAs and sound therapy (265). Now, patients using CIs can also benefit from low-level partial masking sounds (eg, broadband noise, waterfalls, raindrops, etc.) presented in the background (93,94). However, patient preference is varied regarding the type and level of sound. It should be noted that patients will need to wear the CI processor to benefit from the masking sounds, as it is likely the masker will be inaudible when the processor is off.

### Tinnitus Conclusion

While CI candidates and recipients may suffer from tinnitus before and/or after CI surgery, tinnitus relief along with hearing improvement will likely drive patient decision-making toward CI. Clinicians should be prepared to counsel patients on evidence-based postoperative realistic expectations for tinnitus relief with a CI. It would be helpful for the CI clinic to offer tinnitus counseling or refer to a tinnitus counselor for a thorough assessment prior to surgery. Often, simply using the CI can reduce the tinnitus, and for others, background maskers may be effective.

### Summary of CI + HA for Tinnitus Relief

1. Approximately 70%–80% of individuals suffering from tinnitus report improvement following CI (76–79); however, improvement cannot be predicted, so patients should be appropriately counseled regarding realistic expectations and supported with other appropriate therapies if necessary.
2. For some bimodal listeners, it is possible to integrate acoustic hearing with CI stimulation to further reduce troublesome tinnitus (14).

## RECOMMENDATIONS FOR AURAL REHABILITATION

Globally, opportunities and requirements for aural rehabilitation following CI are variable. Some countries have limited, or no, opportunities, while others have an integrated provision within their clinic service model. Provision seems dependent on clinician attitudes, service capacity, funding, and reimbursement (266). Candidacy and access to advanced technology for functional real world benefit is significantly expanding (218,267). CI is now offered to an increasingly wide range of candidates including: elderly prelingual adults/prelingually deaf adults, postlingually deafened adults, and adults with declining cognitive levels.

It is debatable if technology is sufficiently robust for this range of candidates to interpret the electrical, or acoustical and electrical, signal to optimize their auditory potential and functional communication benefits without rehabilitation. A flexible, person-centered aural rehabilitation support service may better realize the economic cost and benefits of implantation. Lack of rehabilitation for some adults may lead to poor outcomes including limited device use, low QOL, limited independence, increased social isolation, loss of communication abilities, and potential cognitive decline. This section focuses on recommendation for consideration of a global implementation of clinician-led individual or group sessions when required and otherwise utilizing e-health online resources, local community, and company opportunities, to deliver cost-effective, flexible rehabilitation.

Several factors affect outcomes following CI including aural rehabilitation and training (268–271). Although some adults (ie, prelingually deafened adults) may not require aural rehabilitation to benefit from CIs, others have shown significant benefit from a structured aural rehabilitation approach (80).

Aural rehabilitation should be a holistic approach that begins prior to surgery and continues until the patient reaches their maximum performance or until their goals are met. Preoperative assessment includes speech and language measures, counseling, establishing person-centered QOL aims, device counseling, and expectation management. Postoperative sessions include counseling through the device acclimatization process, interactive aural rehabilitation sessions (analytic and synthetic exercises), recommending appropriate resources for auditory training at home, and counseling for communication strategies and self-efficacy.

### Measuring Benefit of Aural Rehabilitation

Real life, functional outcomes cannot be sufficiently assessed by speech recognition measures preoperatively and postoperatively. QOL measures provide a more comprehensive insight into real life benefit. Achieving a positive impact on personal QOL, functional sustainable hearing health benefits, communication confidence, and technical self-empowerments constitute a successful outcome and effective intervention (159,272).

One recently developed and validated tool to measure QOL is the Cochlear Implant Quality of Life Profile (CIQOL-35 Profile) and the Cochlear Implant Quality of Life Global (CIQOL-10 Global) (159). The CIQOL-35 Profile is an instrument specifically designed for use with adult CI recipients, which includes 35 items that measure QOL in 6 unidimensional domains (communication, emotional, entertainment, environment, listening effort, and social). The CIQOL-10 Global is a shorter 10-item version providing a single, overall QOL score. These measures can be used to assess QOL in adult CI users at different timepoints preimplantation and postimplantation. The CIQOL measures can be accessed here: https://medicine.musc.edu/departments/otolaryngology/research/cochlear-implant/instruments.

### Managing Expectations Prior to Implantation

Expectation management preimplantation is recommended. Lower (or more reasonable) preoperative expectations is associated with higher QOL outcomes postoperatively (273). A variety of everyday listening situations should be discussed in relation to how they can expect to experience them with their CI and HA. It is often helpful for CI recipients to respond to a list of statements indicating whether they expect to agree or disagree after implantation (eg, “Music will sound natural to me.”). Tinnitus should be included as part of this discussion if applicable. Any statements that suggest inappropriate expectations should be reviewed prior to implantation.

### Recommendations for Aural Rehabilitation

Learning to listen with a CI takes time, as the brain learns to interpret the electrical signal (26). Aural rehabilitation, much like physical therapy, provides activities for adults to detect, listen, and assimilate new sound experiences through latest technology using top-down and bottom-up approaches. Individual cognitive, language, perceptual organization and auditory skills levels are important to consider when designing an aural rehabilitation plan. Some adults benefit from listening with CI alone, using structured aural rehabilitation to gain confidence and adjustment to the electrical signal. Whereas others benefit from opportunities to stream exercises simultaneously to the CI and HA and engage in live voice, real time activities increase communication confidence and binaural summation. As CI recipients progress through their aural rehabilitation plan, they will likely also need scaffolding for navigating more challenging listening situations, which are best supported with binaural hearing such as localization and listening in background noise.

### Group Rehabilitation

Group therapy is a cost-effective way to provide rehabilitation to multiple adults and their communication partners. Communication partners provide peer support, increased social and emotional wellbeing, and increased support for use of technology and assistive technology (268). Group classes can focus on communication strategies, music, device review, accessory review, telephone training, and tinnitus, as examples. Delivery of rehabilitation to groups is increasingly available on videoconferencing and telehealth platforms, which may be appropriate for some patient groups.

### Self-guided Rehabilitation at Home

Online resources provide clinicians with a broad range of free resources aural rehabilitation patients can access at home. These enable adults to practice independently or with a communication partner at any time. Online training has demonstrated improvements in certain auditory skills for adults with CIs (274–277). Exercises encouraging executive functioning skills may improve cognitive skills and higher-order listening skills, such as listening in noise as well; however, results are mixed (80,278). Web- and app-based resources can motivate and engage patients in rehabilitation at home. Structured, free training programs such as SoundSuccess are available globally and can be introduced preoperatively as a baseline and repeated to measure progress over time following CI. For patients without access to the internet, nonstructured activities such as Technology Entertainment Design talks, audio books, and podcasts can be used with subtitles, transcripts, lip-reading or listening alone. Audio books provide an excellent initial acclimatization to listen with a CI because they can follow the text while listening to the spoken story.

### Music-based Rehabilitation

The impact music has on QOL should be considered in holistic hearing healthcare because music continues to matter to many adults, even when the sound quality is initially disappointing. Despite a reduction in listening to music postimplantation, it is rated as highly important to adults with CIs and the second most important acoustical stimulus after speech perception (279,280). The globally available validated Music Related Quality of Life questionnaire identifies individual music rehabilitation needs, measuring music’s impact on QOL and changes in musical experiences postintervention (281).

Free online resources such as Musical Atmospheres and Interactive Music Awareness Program enable independent relearning how to listen to music with opportunities for bimodal musical benefit developed through identifying emotions in music, voice, timbre, and genres through familiar and unfamiliar exercises linked to examples on YouTube (282). While further specifics for music-based rehabilitation are beyond the scope of this guideline, music should not be overlooked as an important aspect to aural rehabilitation and everyday life. Music rehabilitation and training are positive for the elderly in executive function, working memory, episodic memory, and cognitive functioning. Music training has been correlated with better working memory, leading to better structural integrity of the prefrontal cortical areas of the brain (283,284).

### Aural Rehabilitation Conclusions

While there have been no randomized controlled trials (RCTs) investigating the effectiveness of aural rehabilitation and auditory training on outcomes for adult CI recipients, aural rehabilitation can make a significant impact on holistic hearing health and subjective outcomes for adults following CI. A recent investigation of aural rehabilitation in 263 HA users utilizing a placebo-controlled RCT demonstrated no differences in HA outcomes for individuals undergoing rehabilitation via traditional clinician-led programs, at-home auditory training, listening to audiobooks, or an active control group, which was just provided with educational counseling (81). Despite the lack of concrete evidence demonstrating efficacy of aural rehabilitation, there is also no evidence that it would be harmful to a patient’s outcomes and many patients report subjective benefits. Thus, a hybrid sustainable model, utilizing remote e-hearing health service delivery models and in person opportunities potentially enable improved outcomes through the interplay between hearing, technology, and the brain. Aural rehabilitation should be considered within the seamless continuum of holistic hearing health care with acknowledgment of its potential to contribute to the hearing healthcare for healthy aging.

### Summary of Aural Rehabilitation

1. Not all adults require aural rehabilitation, but some have shown significant benefit from a structured aural rehabilitation approach (80). The current literature lacks a RCT to unequivocally evaluate the effectiveness of aural rehabilitation.
2. There are many types of rehabilitation (clinician-led programs, self-guided at-home training, group, etc.) options for patients. Early evidence from HA users suggests that different types of therapy were equally effective (81).
3. CI recipients likely require a personalized aural rehabilitation plan combining remote e-hearing health and in person opportunities to ensure that the therapy meets their goals and is sustainable for the treatment center and patient.

## DISCUSSION

Adults who utilize a bimodal hearing configuration represent a growing patient population, which requires the hearing professional to be knowledgeable in the complexities of the CI and HA systems as well as how they best work in combination. To our knowledge, this is the first comprehensive guideline to be published on bimodal fitting for adult CI users. A review of the literature was completed by a group of experts in the field and compiled to guide professional practice. This guideline was written to address the urgent need for lack of consistent guidelines and awareness of the benefit of bimodal fitting for the treatment of bilateral SNHL in adults. Awareness and guidelines are 2 important steps toward improving access to treatment and ultimately QOL for adults with hearing loss. After extensive review, the most obvious evidence gap in the literature continues to be the fitting of the HA in combination with the CI. Studies to date have shown no effect of fitting formula used; however, further investigation is needed to systematically study varying shapes and degrees of hearing loss (51). Emerging evidence suggests that synchronizing the AGC of the CI and HA offers benefits such as improved SIN recognition and localization (57,58). Further investigation is warranted as these devices become commercially available to patients.

A major limitation to the current guideline is that most of the authors currently practice in the United States, so the recommendations for clinical practice logistics may not be broadly applicable to other parts of the world with unique challenges concerning space, equipment, and patient access to care. Specific barriers to care in other regions were not considered in the development of these guidelines, and further exploration in this area would be of benefit to the field as we continue to expand access to hearing healthcare.

## CONCLUSIONS

The purpose of this document was to provide a guideline for indications for and implementation of bimodal hearing configurations for adults with SNHL. Ultimately, HAs and CIs should be considered part of a hearing loss treatment continuum in which the treating professional is in constant consideration of the optimal device recommendation for each ear, providing binaural amplification whenever possible.

## Data Availability

Data sharing not applicable to this article as no datasets were generated or analyzed during the current study.
